# Targeting C/ **EBP** ****β**** to Suppress Myocardial Fibrosis in Hypertensive Heart Disease: Role of the **ACE2** /Ang‐(1–7) Pathway

**DOI:** 10.1111/jcmm.71139

**Published:** 2026-04-09

**Authors:** Yuanyuan Tie, Zehao Lin, Ming Zhang, Bincheng Ren, Donggang Han, Kexin Li, Ping Jin, Dengfeng Gao

**Affiliations:** ^1^ Department of Cardiology Second Affiliated Hospital of Xi'an Jiaotong University Xi'an China

**Keywords:** ACE2/ang‐(1–7) axis, C/EBP*β*, hypertensive heart disease, myocardial fibrosis

## Abstract

Hypertensive heart disease is characterised by ventricular remodelling and interstitial fibrosis, in which dysregulation of the renin–angiotensin system (RAS) plays a pivotal role. This study investigated whether the transcription factor CCAAT/enhancer‐binding protein *β* (C/EBP*β*) attenuates hypertensive myocardial fibrosis and remodelling by modulating the angiotensin‐converting enzyme 2 (ACE2)/angiotensin‐(1–7) [Ang‐(1–7)] axis. Eight‐week‐old male spontaneously hypertensive rats (SHR) received tail‐vein injection of lentiviral vectors encoding C/EBP*β*, C/EBP*β* short hairpin RNA (shRNA), or negative control (*n* = 6 per group), whereas Wistar–Kyoto rats injected with saline served as normotensive controls. Twelve weeks after injection, blood pressure and echocardiographic parameters were assessed, and cardiac hypertrophy and fibrosis were evaluated by heart weight, heart weight‐to‐body weight ratio, histology, Masson's trichrome staining with quantitative morphometry, immunohistochemistry and Western blotting. Angiotensin II (Ang II) and Ang‐(1–7) levels were quantified in serum and myocardium, and circulating matrix metalloproteinase (MMP)‐2/9, interleukin‐6 (IL‐6) and monocyte chemotactic protein‐1 (MCP‐1) were measured by enzyme‐linked immunosorbent assay (ELISA). In parallel, primary rat cardiac fibroblasts were stimulated with Ang II and treated with lentiviral C/EBP*β* overexpression in combination with ACE2‐specific or control small interfering RNA (siRNA) to examine ACE2‐dependent regulation of collagen I synthesis. C/EBP*β* expression was markedly reduced in SHR myocardium compared with normotensive rats. C/EBP*β* overexpression lowered arterial pressure, improved systolic and diastolic indices, and attenuated left ventricular hypertrophy and cardiomyocyte enlargement. Myocardial fibrosis was reduced, as shown by a smaller collagen‐positive area on Masson's trichrome staining and decreased collagen I, collagen III and transforming growth factor‐*β*1 (TGF‐*β*1) expression. C/EBP*β* overexpression shifted the renin–angiotensin system toward the ACE2/Ang‐(1–7) axis, with higher ACE2 and Ang‐(1–7), lower angiotensin‐converting enzyme (ACE) and Ang II, and reduced IL‐6 and MCP‐1 levels, whereas MMP activity remained largely unchanged. In Ang II–stimulated cardiac fibroblasts, C/EBP*β* upregulated ACE2 and suppressed collagen I, while ACE2 knockdown abolished these antifibrotic effects, supporting an ACE2‐dependent mechanism. In contrast, C/EBP*β* knockdown in vivo had no significant impact on cardiac function, remodelling, fibrosis, inflammation, or RAS components. Myocardial C/EBP*β* expression is reduced in hypertensive rats, and its restoration markedly limits hypertension‐induced cardiac injury. C/EBP*β* overexpression lowers blood pressure, improves systolic and diastolic function and attenuates left ventricular hypertrophy, fibrosis and inflammation. These benefits are associated with a shift from the ACE/Ang II towards the ACE2/Ang‐(1–7) axis and reduced collagen accumulation, supporting C/EBP*β*‐mediated ACE2 activation as a potential therapeutic strategy for hypertensive cardiac remodelling.

## 1 Introduction

Hypertension is a common chronic condition and remains one of the principal modifiable drivers of cardiovascular morbidity and mortality worldwide [1, 2]. Persistent blood pressure elevation induces cardiac structural remodelling with subsequent systolic and/or diastolic dysfunction, changes that are collectively termed hypertensive heart disease. This process can eventually lead to heart failure or sudden cardiac death. Therefore, early blood pressure control and prevention of ventricular remodelling are crucial. Ventricular remodelling is a hallmark pathological feature of hypertensive heart disease, and one of its main manifestations is myocardial interstitial fibrosis.

Hypertensive cardiac remodelling results from the interplay of multiple molecular pathways, including neurohormonal activation, mechanical stress–responsive signalling, oxidative stress and inflammation [3, 4]. Profibrotic mediators such as transforming growth factor‐*β* (TGF‐*β*)/Smad signalling, connective tissue growth factor and endothelin‐1 drive cardiac fibroblast activation and extracellular matrix accumulation, thereby promoting myocardial interstitial fibrosis [5]. Within this network, overactivation of the renin–angiotensin system (RAS) is regarded as a central pathogenic mechanism [6].

The classical arm of the RAS exerts its effects primarily through angiotensin II (Ang II), the principal effector peptide that regulates vascular tone, blood pressure, sodium homeostasis, and hemodynamic stability via activation of Ang II type 1 (AT1) receptors [7]. Beyond these hemodynamic actions, Ang II has potent growth‐promoting and profibrotic properties, stimulating cell proliferation and migration, enhancing collagen synthesis and driving myocardial fibrosis and ventricular remodelling [8]. Consequently, limiting Ang II signalling is a major therapeutic strategy in cardiovascular disease. Angiotensin‐converting enzyme 2 (ACE2) counterbalances the classical RAS axis by catalysing the degradation of Ang II to the vasoprotective peptide angiotensin‐(1–7) [Ang‐(1–7)] [9]. In an acute myocardial infarction rat model, ACE2 overexpression attenuated myocardial fibrosis, reduced angiotensin‐converting enzyme (ACE) and Ang II expression, improved left ventricular remodelling and enhanced cardiac contractile function [10]. These experimental findings suggest that upregulation of ACE2 may also represent a promising therapeutic approach for the treatment of hypertensive heart disease.

CCAAT/enhancer‐binding protein *β* (C/EBP*β*), a transcription factor of the basic leucine zipper family, has been implicated in cardiac structural remodelling [11]. Experimental studies in other cardiac models have shown that C/EBP*β* can exert antiproliferative effects by suppressing CBP/p300‐interacting transactivator with Glu/Asp‐rich carboxy‐terminal domain 4 (CITED4), thereby limiting CITED4‐driven upregulation of the G1/S‐specific cyclin D1 and cardiomyocyte proliferation [12, 13]. Although the C/EBP*β*–CITED4–cyclin D1 axis is not directly examined in the present study, these findings highlight the capacity of C/EBP*β* to modulate cardiomyocyte growth and, more broadly, cardiac remodelling. In our previous work using experimental models of diabetic cardiomyopathy, we found that cardiac C/EBP*β* expression was downregulated in streptozotocin‐induced type 1 diabetic mice and in high‐glucose–treated neonatal cardiomyocytes and cardiac fibroblasts, whereas lentiviral C/EBP*β* overexpression increased C/EBP*β* binding to the ACE2 promoter, upregulated ACE2 expression, and significantly reduced collagen deposition and cardiomyocyte apoptosis [14]. Given the central role of ACE2 in counterbalancing the classical RAS and limiting collagen accumulation, we hypothesised that C/EBP*β* overexpression may attenuate myocardial fibrosis and ventricular remodelling in hypertensive heart disease by enhancing ACE2 expression and thereby suppressing collagen synthesis.

## 2 Materials and Methods

### 2.1 Animal Protocol

All animal experiments were conducted in accordance with the U.S. National Institutes of Health (NIH) Guide for the Care and Use of Laboratory Animals. Eight‐week‐old male Wistar–Kyoto rats (WKY) and spontaneously hypertensive rats (SHR) were housed at 22°C under a 12‐h light/dark cycle. After an 8‐week acclimatisation period and blood pressure monitoring, systolic blood pressure in all SHR rats exceeded 160 mmHg, confirming the successful establishment of the hypertensive model. The rats were then randomly allocated into four groups (*n* = 6 per group): (1) WKY rats treated with saline (WKY + Saline), (2) SHR rats receiving a negative‐control short hairpin RNA (shRNA) lentiviral vector (SHR + LV‐NC), (3) SHR rats receiving a C/EBP*β* shRNA lentiviral vector (SHR + LV‐shC/EBP*β*) and (4) SHR rats receiving a C/EBP*β* overexpression lentiviral vector (SHR + LV‐C/EBP*β*). SHR rats in the three indicated groups received a tail‐vein injection of 1 × 10^8 transducing units (TU)/50 μL of lentiviral vector containing sh‐NC, sh‐C/EBP*β* or C/EBP*β* (GenePharma, Shanghai, China). WKY rats treated with saline served as normotensive baseline controls. The mechanistic effects of C/EBP*β* modulation were evaluated by comparing the three SHR groups, which all shared the same lentiviral backbone, with SHR + LV‐NC as the empty‐vector control. Twelve weeks after vector or saline injection, all rats were euthanised.

### 2.2 Echocardiography and Measurement of Blood Pressure

At week 20, prior to the euthanasia of all the rats, transthoracic parasternal echocardiography was performed to assess cardiac structure and function using a Vevo 770 imaging system (VisualSonics, Toronto, ON, Canada). Left ventricular parameters were assessed by M‐mode echocardiography according to the American Society of Echocardiography guidelines, including left ventricular ejection fraction (LVEF), fractional shortening (FS), left ventricular end‐diastolic diameter (LVEDd) and left ventricular posterior wall thickness at end‐diastole (LVPWd) [15]. We also evaluated the peak velocity of ventricular filling, including peak E wave, peak A wave and the E‐to‐A (E/A) ratio using pulsed‐wave Doppler echocardiography. Echocardiographic measurements were analysed offline by an experienced investigator who was blinded to the group allocation. All vital signs, including systolic blood pressure (SBP), diastolic blood pressure (DBP) and heart rate (HR), were measured using a noninvasive tail‐cuff system (Softron BP‐98A; Softron, Tokyo, Japan).

### 2.3 Histology and Immunohistochemistry

Rat hearts fixed in 4% paraformaldehyde were embedded in paraffin after dehydration in a graded ethanol series and sectioned at 5 μm. Cardiomyocyte cross‐sectional area was measured on haematoxylin and eosin (H&E)‐stained sections. Myocardial fibrosis was assessed on Masson's trichrome‐stained sections (Solarbio, Beijing, China), in which collagen fibres were stained blue and cardiomyocytes were stained red. The extent of fibrosis was quantified as the collagen‐positive area divided by the total myocardial area and expressed as a percentage. Paraffin sections were prepared in the same manner for immunohistochemistry (IHC). The slides were incubated overnight at 4°C with primary antibodies, namely anti‐C/EBP*β*, anti‐ACE2, anti‐ACE, anti‐collagen I, anti‐collagen III and anti‐transforming growth factor‐*β*1 (TGF‐*β*1) (all from Abcam, Cambridge, MA, USA), followed by incubation with the corresponding secondary antibody for 30 min at 37°C. Stained sections were examined at 400× magnification using Image‐Pro Plus image‐analysis software (version 5.0; Media Cybernetics, Houston, TX, USA). Histological sections were evaluated by an independent observer who was blinded to the experimental groups.

### 2.4 Elisa

Blood samples were collected from the abdominal aorta of anaesthetised rats and allowed to clot at room temperature for 30 min. Subsequently, the samples were centrifuged at 3000 × g for 15 min at 4°C to obtain serum. The supernatant was carefully aliquoted and stored at −80°C until analysis. The levels of Ang‐(1–7) (Elabscience, Wuhan, China), Ang II, matrix metalloproteinase (MMP)‐2, MMP‐9, interleukin‐6 (IL‐6) and monocyte chemotactic protein‐1 (MCP‐1) (all from R&D Systems, Quantikine ELISA, Minneapolis, MN, USA) in the serum were measured according to the manufacturers' instructions.

Left ventricular myocardial tissue samples were homogenised on ice in pre‐chilled radioimmunoprecipitation assay (RIPA) lysis buffer containing 1 mM phenylmethylsulfonyl fluoride (PMSF) and a protease inhibitor cocktail to prevent protein degradation. The homogenate‐to‐buffer ratio was maintained at 1:9 (weight: volume). The homogenates were then incubated on ice for 2 h with intermittent vortexing to ensure complete protein extraction, followed by centrifugation at 12,000 × g for 20 min at 4°C. The clear supernatant was collected as the total tissue protein lysate. The total protein concentration of each lysate was determined using a bicinchoninic acid (BCA) protein assay kit with bovine serum albumin as the standard. Ang II and Ang‐(1–7) levels in the myocardial lysates were measured using the same ELISA kits as for the serum.

### 2.5 Gelatin Zymography

The activities of matrix metalloproteinases (MMP‐2 and MMP‐9) in myocardial tissue were determined by gelatin zymography. Equal amounts of protein samples were mixed with nonreducing sodium dodecyl sulphate (SDS) sample buffer and separated by electrophoresis on 10% SDS–polyacrylamide gels containing 0.1% (w/v) gelatin as the substrate. Following electrophoresis, the gels were washed twice in 2.5% Triton×‐100 to remove SDS and allow enzyme renaturation, and subsequently equilibrated in washing buffer (50 mM Tris–HCl, pH 7.5, 100 mM NaCl). The gels were then incubated in activation buffer (50 mM Tris–HCl, pH 7.5, 150 mM NaCl, 10 mM CaCl_2_ and 1 μM ZnCl_2_) at 37°C for 12–24 h to facilitate gelatin digestion. After incubation, the gels were stained with Coomassie Brilliant Blue R‐250 and destained until clear lytic bands corresponding to proteolytic zones became visible. The gelatinolytic bands at approximately 72 kDa and 92 kDa were identified as MMP‐2 and MMP‐9 activities respectively. Band intensities were quantified using ImageJ image‐analysis software (National Institutes of Health, Bethesda, MD, USA) for densitometric analysis.

### 2.6 Western Blot

Proteins were extracted from myocardial tissue using a total protein extraction kit (Beitian Biotechnology, Jiangsu, China) according to the manufacturer's instructions. Equal amounts of protein were separated by 10%–12% sodium dodecyl sulphate–polyacrylamide gel electrophoresis (SDS‐PAGE) and then transferred onto polyvinylidene difluoride (PVDF) membranes (Millipore, Eschborn, Germany). The membranes were blocked with 5% (*w/v*) skim milk at room temperature for 2 h and subsequently incubated overnight at 4°C on a shaker with the following primary antibodies: anti‐C/EBP*β* (Abcam, Cambridge, MA, USA; Santa Cruz Biotechnology, Santa Cruz, CA, USA), anti‐ACE2, anti‐collagen I and anti‐collagen III (all from Abcam). After three washes with Tris‐buffered saline containing 0.1% Tween‐20 (TBST), the membranes were incubated with the appropriate horseradish peroxidase‐conjugated secondary antibodies at room temperature for 90 min. *β*‐Tubulin (Proteintech Group, Wuhan, Hubei, China) was used as a loading control. Protein bands were visualised using an Amersham Imager 600 (Fairfield, CT, USA) and quantified by densitometric analysis with Adobe Photoshop CS6 (San Jose, CA, USA).

### 2.7 Cell Culture

Primary rat cardiac fibroblasts were obtained from Xi'an Huimei Biotechnology Co. Ltd. (Xi'an, China). Cells were cultured in Dulbecco's modified Eagle's medium (DMEM; Thermo Fisher Scientific, Waltham, MA, USA) supplemented with 10% foetal bovine serum and maintained at 37°C in a humidified incubator with 5% CO_2_. When cell confluence reached approximately 70%, fibroblasts were randomly allocated to three groups and subjected to the following treatments: (1) negative control group treated with negative control small interfering RNA (siRNA) (LV‐NC + si‐NC); (2) C/EBP*β* overexpression combined with ACE2‐specific siRNA (LV‐C/EBP*β*+si‐ACE2) and (3) C/EBP*β* overexpression combined with negative control siRNA (LV‐C/EBP*β*+si‐NC). Lentiviral vectors and siRNAs (GenePharma, Shanghai, China) were added to the culture medium and co‐incubated with the cells for 24 h according to the manufacturer's instructions. The medium was then replaced with fresh complete culture medium containing angiotensin II (1 μM), and cells were further stimulated for 48 h to induce collagen synthesis [16, 17]. At the end of the treatment, cells were harvested, and total protein was extracted for Western blot analysis of C/EBP*β*, ACE2 and collagen I expression.

### 2.8 Statistical Analysis

Results are expressed as the mean ± standard deviation (SD) from at least three independent experiments. For comparisons between two groups, an independent‐samples *t*‐test was used. For comparisons among more than two groups, group differences were analysed using one‐way analysis of variance (ANOVA) followed by Fisher's least significant difference (LSD) test for post hoc multiple comparisons. The assumption of homogeneity of variances was tested and was satisfied for all datasets. A *p* < 0.05 was considered statistically significant. Statistical analyses were performed using SPSS software, version 17.0 (SPSS Inc., Chicago, IL, USA).

## 3 Result

### 3.1 Blood Pressure Decreased Significantly in the C/EBP*β* Overexpression Group

**FIGURE 1:**
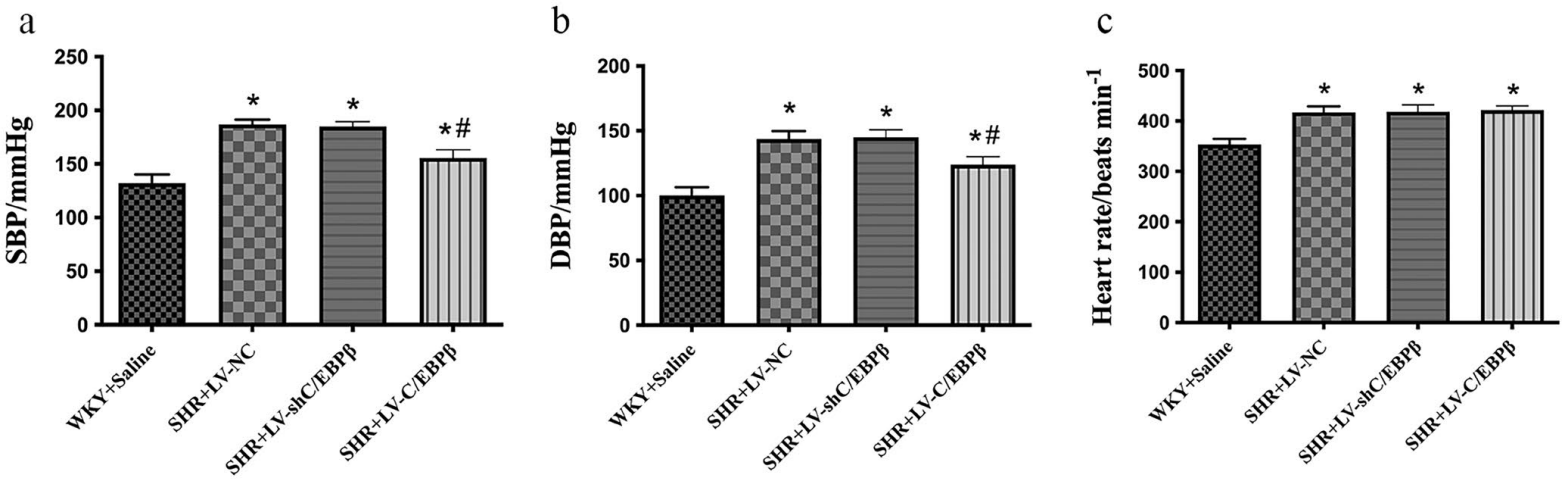
Blood pressure and heart rate in experimental rats. (a) Systolic blood pressure (SBP). (b) Diastolic blood pressure (DBP). (c) Heart rate (HR). WKY + Saline: Wistar Kyoto rats (normotensive control). SHR + LV‐NC: Spontaneously hypertensive rats injected with negative control lentivirus. SHR + LV‐shC/EBP*β*: SHR rats with lentiviral knockdown of C/EBP*β*. SHR + LV‐C/EBP*β*: SHR rats with lentiviral overexpression of C/EBP*β*. Data are expressed as the mean ± standard deviation (SD); *n* = 6 rats per group. Statistical analysis was performed using one‐way analysis of variance (ANOVA) followed by Fisher's least significant difference (LSD) post hoc test. *p < 0.05 versus WKY + Saline; ^#^ *p < 0.05* versus SHR + LV‐NC.

Blood pressure and heart rate were measured using a noninvasive tail‐cuff system. SHR rats showed significantly higher blood pressure and heart rate compared with the WKY control group. In the C/EBP*β* overexpression group, blood pressure was significantly reduced compared with the SHR + LV‐NC group (*p* < 0.05; Table 1 and Figure 1). However, no statistically significant difference in blood pressure was detected between the SHR + LV‐NC and SHR + LV‐shC/EBP*β* groups (*p* > 0.05; Table 1 and Figure 1). Similarly, heart rate did not differ significantly among the SHR groups (*p* > 0.05; Table 1 and Figure 1).

**TABLE 1:** Blood pressure, heart rate and HW/BW in experimental rat groups.

Parameters	WKY + saline	SHR		
		LV‐NC	LV‐shC/EBP*β*	LV‐C/EBP*β*
SBP (mmHg)	132.27 ± 7.74	187.00 ± 4.35*	184.96 ± 4.52*	155.72 ± 7.59* ^,^ **
DBP (mmHg)	100.06 ± 6.30	143.57 ± 6.24*	145.11 ± 5.70*	123.98 ± 6.14* ^,^ **
HR (bpm)	353.6 ± 10.81	417.4 ± 11.72*	418.4 ± 13.61*	422 ± 7.78*
HW/BW (mg/g)	2.62 ± 0.02	4.07 ± 0.10*	4.08 ± 0.09*	3.40 ± 0.03* ^,^ **

*Note:* Data are expressed as the mean ± standard deviation (SD); *n* = 6 rats per group. Statistical analysis was performed using one‐way analysis of variance (ANOVA) followed by Fisher's least significant difference (LSD) post hoc test.

Abbreviations: DBP, diastolic blood pressure; HR, heart rate; HW/BW, quantitative analysis of the heart weight‐to‐body weight; KY + Saline, Wistar Kyoto rats (normotensive control); SBP, Systolic blood pressure; SHR + LV‐C/EBP*β*, SHR rats with lentiviral overexpression of C/EBP*β*; SHR + LV‐NC, spontaneously hypertensive rats injected with negative control lentivirus; SHR + LV‐shC/EBP*β*, SHR rats with lentiviral knockdown of C/EBP*β*.

* *p* < 0.05 versus WKY + Saline.

** *p* < 0.05 versus SHR + LV‐NC.

### 3.2 Overexpression of C/EBP*β* Significantly Improves Cardiac Function in SHR Rats

**FIGURE 2:**
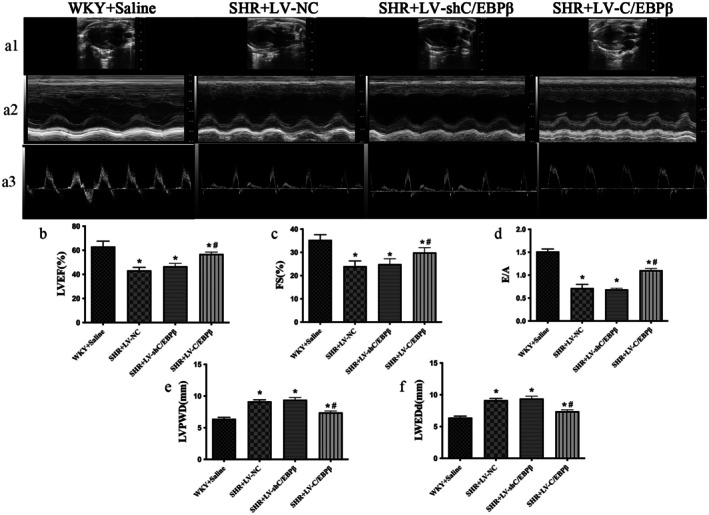
Echocardiographic assessment of cardiac function in experimental rats. (a1, a2) Representative two‐dimensional and M‐mode echocardiograms. (a3) Representative pulsed‐wave Doppler recordings of mitral inflow. (b) Left ventricular ejection fraction (LVEF). (c) Fractional shortening (FS). (d) Ratio of early to late mitral inflow velocity (E/A). (e) Left ventricular posterior wall thickness at end‐diastole (LVPWd). (f) Left ventricular end‐diastolic dimension (LVEDd). WKY + Saline: Wistar Kyoto rats (normotensive control). SHR + LV‐NC: Spontaneously hypertensive rats injected with negative control lentivirus. SHR + LV‐shC/EBP*β*: SHR rats with lentiviral knockdown of C/EBP*β*. SHR + LV‐C/EBP*β*: SHR rats with lentiviral overexpression of C/EBP*β*. Data are expressed as the mean ± standard deviation (SD); *n* = 6 rats per group. Statistical analysis was performed using one‐way analysis of variance (ANOVA) followed by Fisher's least significant difference (LSD) post hoc test. *p < 0.05 versus WKY + Saline; ^#^ *p < 0.05* versus SHR + LV‐NC.

Compared with the normal control group, SHR rats exhibited a significant reduction in cardiac function. M‐mode echocardiography revealed cardiac chamber enlargement, a notable increase in LVEDd, reduced myocardial motion and reduced contractility. The data indicated a significant decrease in LVEF and FS, suggesting a marked decline in systolic function in SHR rats. Additionally, LVPWd was significantly increased, and the E/A ratio measured at the four‐chamber view was reduced, indicating impaired diastolic function. Overexpression of C/EBP*β*, compared with the empty vector group, significantly increased LVEF and FS, indicating an improvement in cardiac contractility. It also reduced LVPWd and LVEDd, enhanced myocardial compliance and restored the E/A ratio, suggesting that C/EBP*β* overexpression effectively alleviates both systolic and diastolic dysfunction induced by hypertension (*p* < 0.05; Figure 2). In contrast, lentiviral‐mediated C/EBP*β* knockdown did not significantly alter LVEF, FS, or E/A, nor did it impact LVEDd and LVPWd (*p* > 0.05; Figure 2). These findings indicate that lentiviral‐mediated C/EBP*β* knockdown does not significantly affect cardiac function in hypertensive heart disease.

### 3.3 Overexpression of C/EBP*β* Relieves Myocardial Hypertrophy in SHR Rats

**FIGURE 3:**
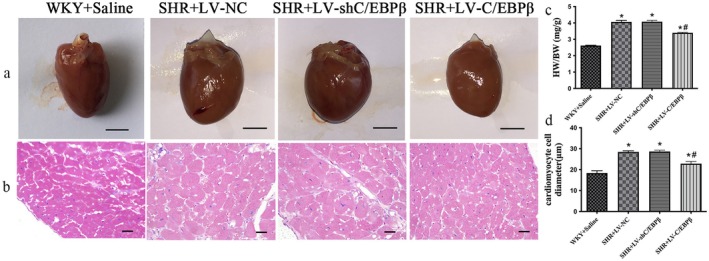
Histological and morphological analysis of cardiac hypertrophy in experimental rats. (a) Representative gross morphology of hearts from each group (scale bar = 5 mm). (b) Representative haematoxylin and eosin (H&E) staining of left ventricular sections (original magnification, ×400; scale bar = 20 μm). (c) Quantitative analysis of the heart weight‐to‐body weight (HW/BW). (d) Quantitative analysis of cardiomyocyte cross‐sectional diameter. WKY + Saline: Wistar Kyoto rats (normotensive control). SHR + LV‐NC: Spontaneously hypertensive rats injected with negative control lentivirus. SHR + LV‐shC/EBP*β*: SHR rats with lentiviral knockdown of C/EBP*β*. SHR + LV‐C/EBP*β*: SHR rats with lentiviral overexpression of C/EBP*β*. Data are expressed as the mean ± standard deviation (SD); *n* = 6 rats per group. Statistical analysis was performed using one‐way analysis of variance (ANOVA) followed by Fisher's least significant difference (LSD) post hoc test. *p < 0.05 versus WKY + Saline; ^#^ *p < 0.05* versus SHR + LV‐NC.

The heart weight (HW) of each rat was measured, and the heart weight‐to‐body weight (HW/BW) was calculated. Compared with the empty vector control group, both HW and HW/BW were significantly reduced in the C/EBP*β* overexpression group (*p* < 0.05; Table 1 and Figure 3c), while there was no significant difference between the C/EBP*β* knockdown group and the empty vector control group (*p* > 0.05; Table 1 and Figure 3c). At the end of the experiment, gross examination of the hearts showed that, compared with the normal control group, hearts from SHR rats in all experimental groups were enlarged to varying degrees, with a rounded and blunted apex and increased stiffness. The increase was particularly pronounced in the empty vector control group and the C/EBP*β* knockdown group, while overexpression of C/EBP*β* partially attenuated these alterations (*p* < 0.05; Figure 3a). At 400× magnification, cross‐sectional images revealed that cardiomyocytes in normal rats were uniform in size, regularly arranged and evenly stained, whereas cardiomyocytes in SHR rats appeared markedly hypertrophic, irregularly arranged and unevenly stained (Figure 3b). Compared with the empty vector group, the C/EBP*β* overexpression group exhibited smaller cardiomyocyte diameters and a relatively more orderly arrangement (*p* < 0.05; Figure 3d), whereas no significant differences were observed in the C/EBP*β* knockdown group (*p* > 0.05; Figure 3d).

### 3.4 Overexpression of C/EBP*β* Inhibits Myocardial Fibrosis in SHR Rats

**FIGURE 4:**
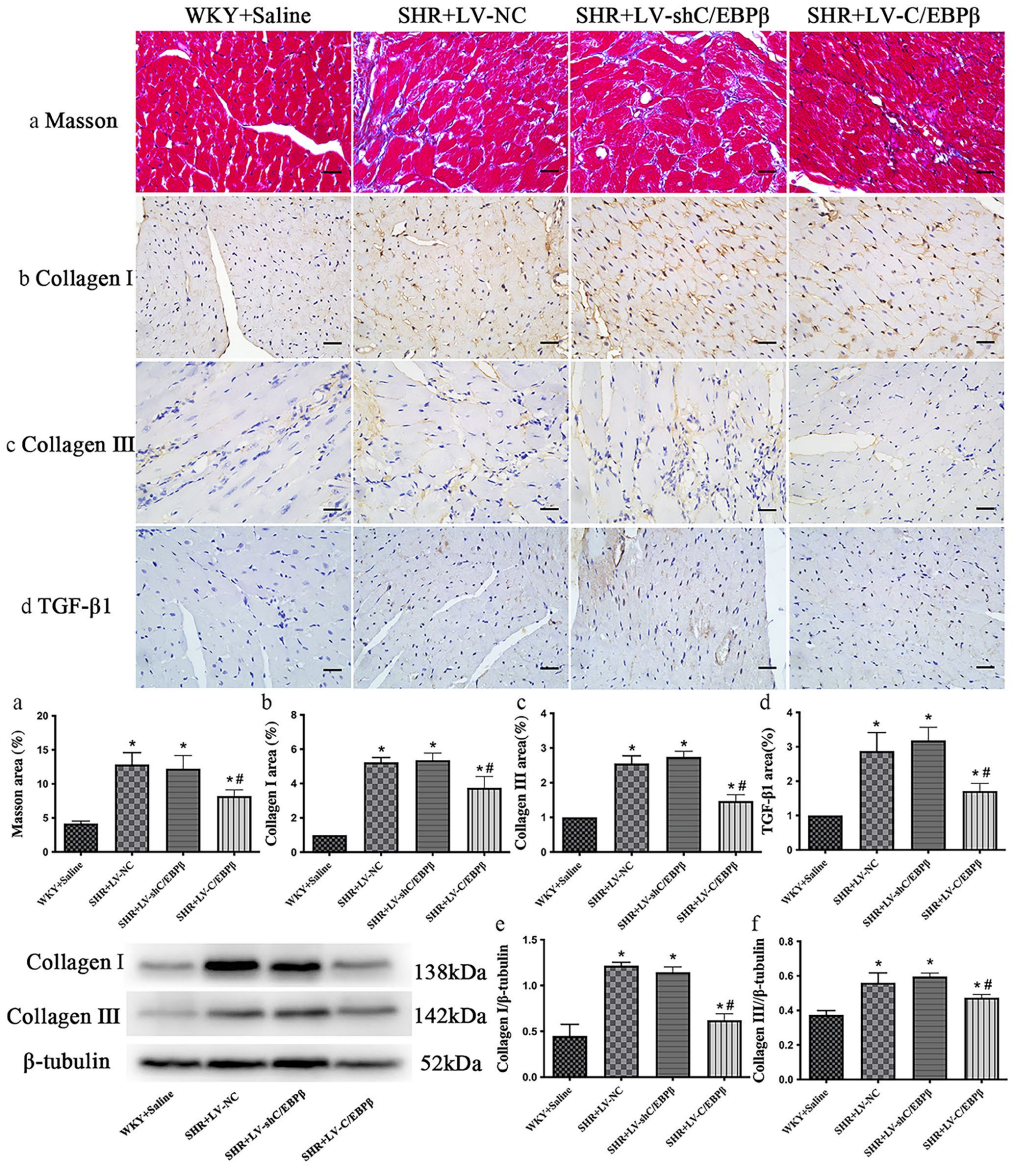
Cardiac fibrosis and collagen expression in experimental rats. (a) Masson's trichrome staining of myocardial sections (first row; scale bar: 20 μm). (b–d) Immunohistochemical staining and quantitative analysis of collagen I (b, second row; scale bar: 20 μm), collagen III (c, third row; scale bar: 20 μm) and transforming growth factor‐*β*1 (TGF‐*β*1) expression (d, fourth row; scale bar: 20 μm). (e, f) Representative Western blots and quantitative analysis of collagen I (e) and collagen III (f). *β*‐tubulin was used as the loading control. WKY + Saline: Wistar Kyoto rats (normotensive control). SHR + LV‐NC: Spontaneously, hypertensive rats were injected with a negative control lentivirus. SHR + LV‐shC/EBP*β*: SHR rats with lentiviral knockdown of C/EBP*β*. SHR + LV‐C/EBP*β*: SHR rats with lentiviral overexpression of C/EBP*β*. Data are expressed as the mean ± standard deviation (SD); *n* = 6 rats per group. Statistical analysis was performed using one‐way analysis of variance (ANOVA) followed by Fisher's least significant difference (LSD) post hoc test. *p < 0.05 versus WKY + Saline; ^#^ *p < 0.05* versus SHR + LV‐NC.

Masson's trichrome staining demonstrated that total myocardial collagen content was significantly increased in SHR + LV‐NC rats compared with WKY + Saline rats. Collagen content did not differ between SHR + LV‐shC/EBP*β* and SHR + LV‐NC rats, whereas SHR + LV‐C/EBP*β* rats exhibited a markedly smaller collagen‐positive area than SHR + LV‐NC rats (*p* < 0.05; Figure 4a), indicating that C/EBP*β* overexpression attenuated hypertensive myocardial fibrosis.

In parallel, IHC staining for collagen I, collagen III and TGF‐*β*1 was performed. In the WKY + Saline group, only a small amount of brownish linear or punctate staining corresponding to collagen I or collagen III was observed in the myocardial interstitial and perivascular regions. Compared with WKY + Saline rats, SHR + LV‐NC rats showed markedly stronger staining in these regions, indicating increased collagen I and collagen III deposition. By contrast, SHR + LV‐C/EBP*β* rats displayed significantly reduced collagen I, collagen III and TGF‐*β*1 staining relative to SHR + LV‐NC rats (*p* < 0.05; Figure 4b–d), whereas no significant differences were observed between SHR + LV‐shC/EBP*β* and SHR + LV‐NC rats (*p* > 0.05; Figure 4b–d).

Western blot analysis further corroborated the histological findings. Collagen I and collagen III protein levels in myocardial tissue were significantly higher in SHR + LV‐NC rats than in WKY + Saline rats, while C/EBP*β* overexpression in SHR + LV‐C/EBP*β* rats led to a significant decrease in both collagen I and collagen III expression compared with SHR + LV‐NC rats (*p* < 0.05; Figure 4e,f). No significant differences were detected between SHR + LV‐shC/EBP*β* and SHR + LV‐NC rats (*p* > 0.05; Figure 4e,f).

**FIGURE 5:**
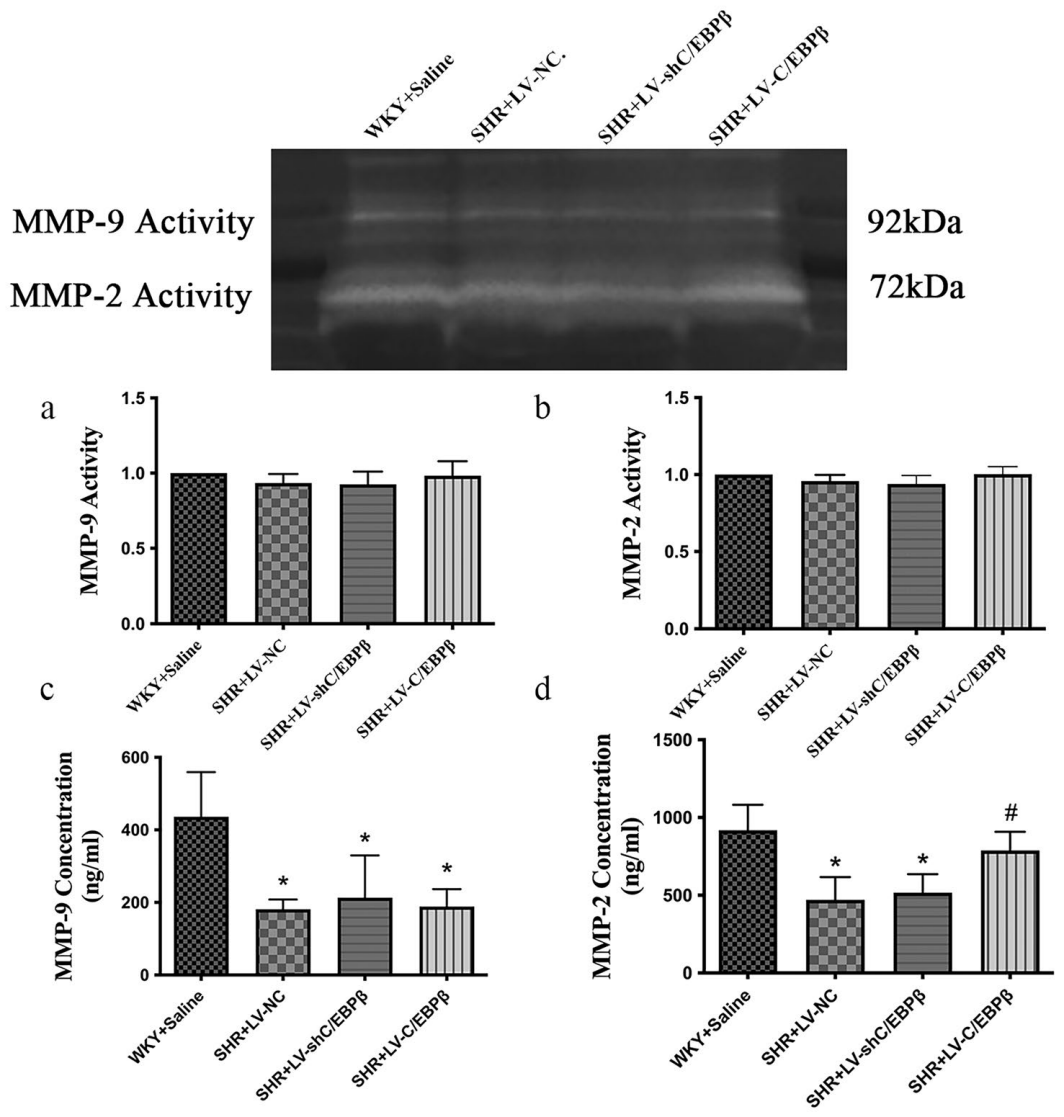
Expression and activity of MMP‐2 and MMP‐9 in experimental rats. (a, b) Gelatin zymography of myocardial matrix metalloproteinase‐9 (MMP‐9) and matrix metalloproteinase‐2 (MMP‐2) activities. (c, d) Serum MMP‐9 and MMP‐2 levels were measured by enzyme‐linked immunosorbent assay (ELISA). WKY + Saline: Wistar Kyoto rats (normotensive control). SHR + LV‐NC: Spontaneously hypertensive rats injected with negative control lentivirus. SHR + LV‐shC/EBP*β*: SHR rats with lentiviral knockdown of C/EBP*β*. SHR + LV‐C/EBP*β*: SHR rats with lentiviral overexpression of C/EBP*β*. Data are expressed as the mean ± standard deviation (SD); *n* = 6 rats per group. Statistical analysis was performed using one‐way analysis of variance (ANOVA) followed by Fisher's least significant difference (LSD) post hoc test. *p < 0.05 versus WKY + Saline; ^#^ *p < 0.05* versus SHR + LV‐NC.

ELISA quantification of serum MMP‐2 and MMP‐9 levels revealed that the C/EBP*β* overexpression group significantly increased the concentration of MMP‐2 compared with the empty vector control group, showing a result comparable to the normal control group (*p* < 0.05; Figure 5d). In contrast, no statistically significant difference was observed between the empty vector group and the C/EBP*β* knockdown group (*p* > 0.05; Figure 5d). Furthermore, the levels of MMP‐9 in all three SHR rat groups were significantly reduced compared to the normal control group (*p* < 0.05; Figure 5c), with no statistically significant differences detected among the three experimental groups (*p* > 0.05; Figure 5c). Gelatin zymography assays for MMP‐2 and MMP‐9 activities showed no significant differences across the groups (*p* > 0.05; Figure 5a,b).

### 3.5 Overexpression of C/EBP*β* Inhibits Inflammatory Response in SHR Rats

**FIGURE 6:**
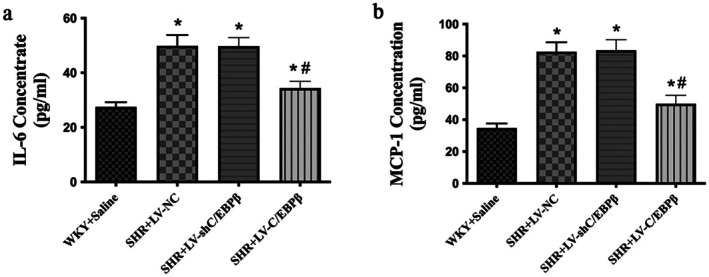
Serum inflammatory cytokine levels in experimental rats. (a) Serum interleukin‐6 (IL‐6) and (b) monocyte chemoattractant protein‐1 (MCP‐1) levels, measured using enzyme‐linked immunosorbent assay (ELISA). WKY + Saline: Wistar Kyoto rats (normotensive control). SHR + LV‐NC: Spontaneously hypertensive rats injected with negative control lentivirus. SHR + LV‐shC/EBP*β*: SHR rats with lentiviral knockdown of C/EBP*β*. SHR + LV‐C/EBP*β*: SHR rats with lentiviral overexpression of C/EBP*β*. Data are expressed as the mean ± standard deviation (SD); *n* = 6 rats per group. Statistical analysis was performed using one‐way analysis of variance (ANOVA) followed by Fisher's least significant difference (LSD) post hoc test. *p < 0.05 versus WKY + Saline; ^#^ *p < 0.05* versus SHR + LV‐NC.

Serum levels of key inflammatory cytokines were measured using ELISA. The results revealed a significant increase in IL‐6 and MCP‐1 levels in the empty vector control group compared to the normal control group (*p* < 0.05; Figure 6a,b). In contrast, C/EBP*β* overexpression notably reduced the levels of IL‐6 and MCP‐1 compared to the empty vector control group (*p* < 0.05; Figure 6a,b), while no significant differences were observed in the C/EBP*β* knockdown group compared to the empty vector control group (*p* > 0.05; Figure 6a,b). These findings suggest that C/EBP*β* overexpression attenuates hypertension‐induced inflammatory responses.

### 3.6 C/EBP*β* Expression Is Reduced in SHR Rats

**FIGURE 7:**
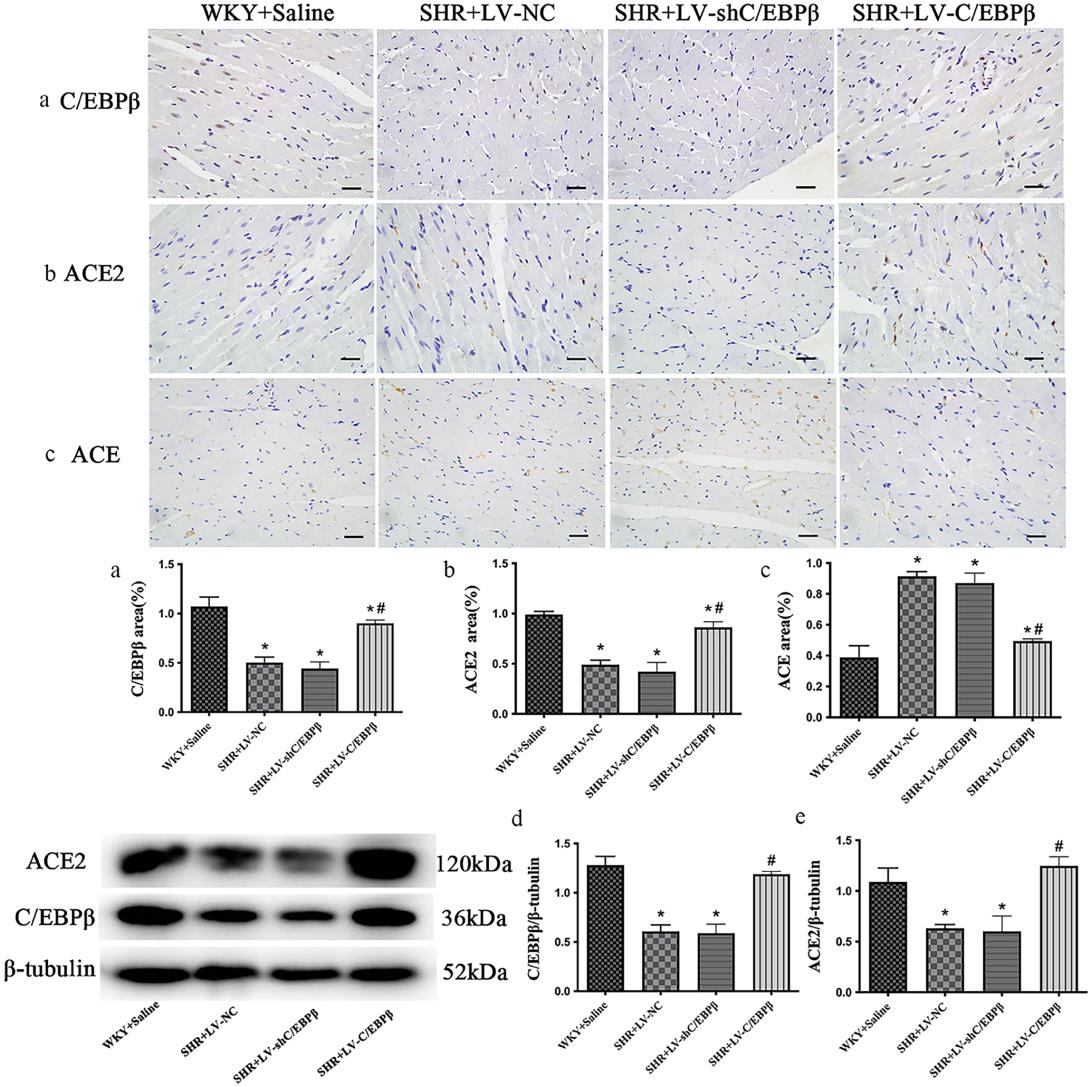
C/EBP*β*, ACE2 and ACE expression in myocardial tissue. (a–c) Representative immunohistochemical staining of CCAAT/enhancer‐binding protein *β* (C/EBP*β*), angiotensin‐converting enzyme 2 (ACE2) and angiotensin‐converting enzyme (ACE) in myocardial sections from each group. (d, e) Representative Western blots and quantitative analysis of C/EBP*β* (d) and ACE2 (e) protein levels in heart tissue. *β*‐tubulin was used as the loading control. WKY + Saline: Wistar Kyoto rats (normotensive control). SHR + LV‐NC: Spontaneously hypertensive rats injected with negative control lentivirus. SHR + LV‐shC/EBP*β*: SHR rats with lentiviral knockdown of C/EBP*β*. SHR + LV‐C/EBP*β*: SHR rats with lentiviral overexpression of C/EBP*β*. Data are expressed as the mean ± standard deviation (SD); *n* = 6 rats per group. Statistical analysis was performed using one‐way analysis of variance (ANOVA) followed by Fisher's least significant difference (LSD) post hoc test. *p < 0.05 versus WKY + Saline; ^#^ *p* < 0.05 versus SHR + LV‐NC.

Western blotting and immunohistochemistry showed that myocardial C/EBP*β* expression was significantly lower in the SHR empty‐vector control group than in normal controls (*p* < 0.05; Figure 7a,d). Lentiviral C/EBP*β* overexpression markedly increased C/EBP*β* levels relative to the empty‐vector group (*p* < 0.05; Figure 7a,d). In contrast, C/EBP*β* shRNA did not further reduce C/EBP*β* expression compared with the empty‐vector group (*p* > 0.05; Figure 7a,d), probably because baseline C/EBP*β* levels in hypertensive myocardium were already low. This “floor effect” may at least partly account for the lack of overt changes in cardiac function, fibrosis or inflammatory markers in the C/EBP*β* knockdown group.

### 3.7 Levels of the ACE2 and ACE Expression in SHR Myocardium

Consistent findings from Western blot and IHC analyses demonstrated that ACE2 expression in the myocardial tissue of rats in the SHR empty vector control group was significantly lower than that in the normal control group. Compared to the empty vector control group, C/EBP*β* overexpression significantly increased ACE2 expression (*p* < 0.05; Figure 7b,e). However, there was no significant difference in ACE2 expression in the C/EBP*β* knockdown group compared with the empty vector control group (*p* > 0.05; Figure 7b,e). In SHR rats, the expression level of ACE was significantly elevated. Particularly, both the empty vector control group and the C/EBP*β* knockdown group showed high ACE expression, with no statistically significant difference observed between these two groups. In contrast, the C/EBP*β* overexpression group exhibited a marked reduction in ACE expression compared with the empty vector control group (*p* < 0.05; Figure 7c).

### 3.8 Effect on Ang II and Ang‐(1–7) Expression Levels

**FIGURE 8:**
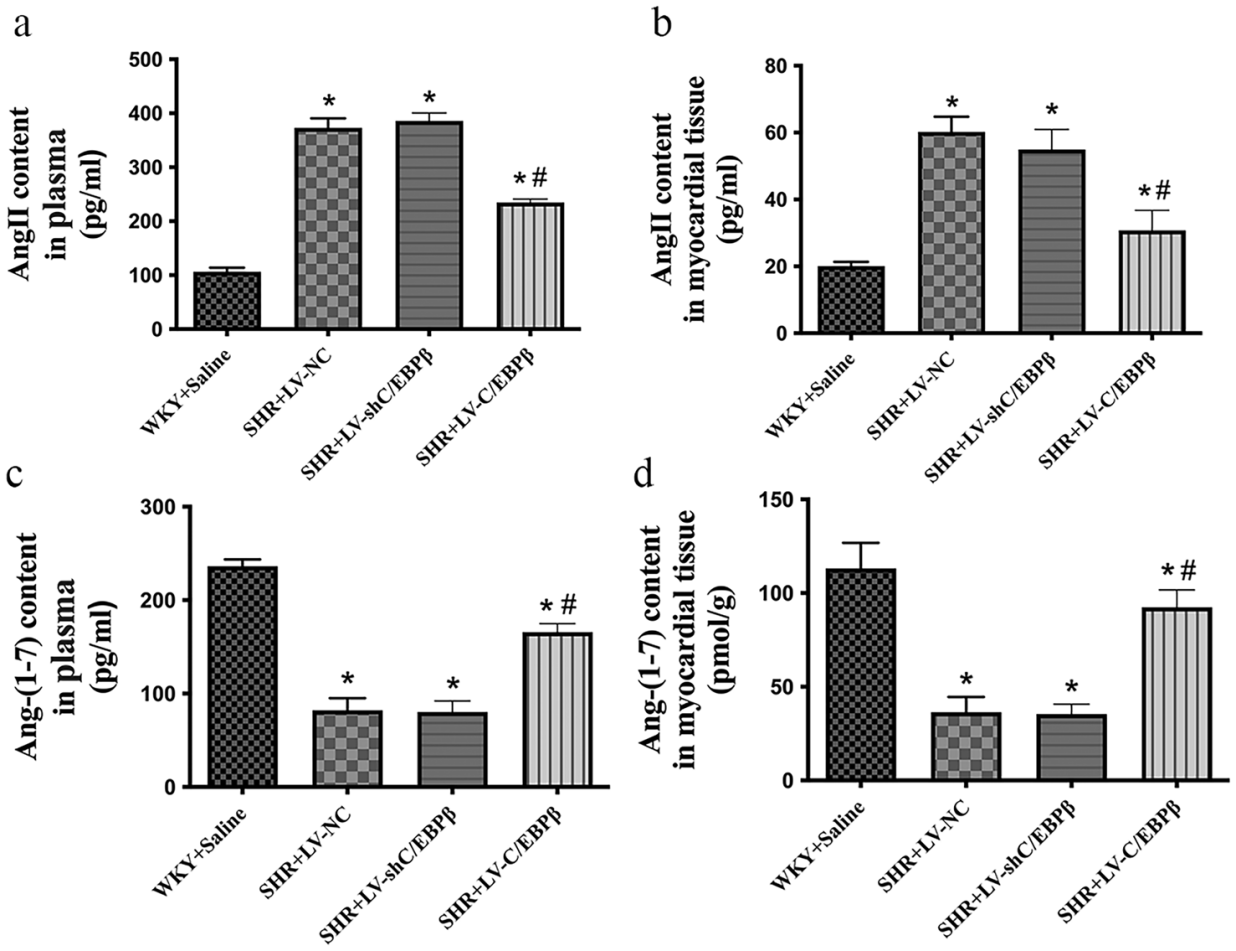
Angiotensin levels in myocardial tissue and serum. Serum and myocardial concentrations of angiotensin II (Ang II) (a, b) and angiotensin‐(1–7) [Ang‐(1–7)] (c, d) were measured using enzyme‐linked immunosorbent assay (ELISA). WKY + Saline: Wistar Kyoto rats (normotensive control). SHR + LV‐NC: Spontaneously hypertensive rats injected with negative control lentivirus. SHR + LV‐shC/EBP*β*: SHR rats with lentiviral knockdown of C/EBP*β*. SHR + LV‐C/EBP*β*: SHR rats with lentiviral overexpression of C/EBP*β*. Data are expressed as the mean ± standard deviation (SD); *n* = 6 rats per group. Statistical analysis was performed using one‐way analysis of variance (ANOVA) followed by Fisher's least significant difference (LSD) post hoc test. *p < 0.05 versus WKY + Saline; ^#^ *p* < 0.05 versus SHR + LV‐NC.

ELISA was performed to quantify Ang II and Ang‐(1–7) levels in myocardial tissue and serum. Compared with the normal control group, the SHR empty vector control group showed significantly increased Ang II levels and decreased Ang‐(1–7) levels in both myocardial tissue and serum (*p* < 0.05; Figure 8). Compared with the empty vector control group, the C/EBP*β* overexpression group exhibited significantly reduced Ang II levels and increased Ang‐(1–7) levels in both myocardial tissue and serum (*p* < 0.05; Figure 8). In contrast, the C/EBP*β* knockdown group did not significantly alter either Ang II or Ang‐(1–7) levels relative to the empty vector control group (*p* > 0.05; Figure 8).

### 3.9 C/EBP*β* Attenuates Collagen Synthesis Through ACE2 Upregulation

**FIGURE 9:**
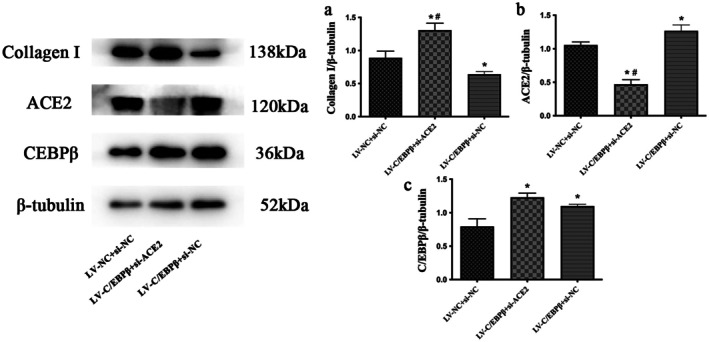
C/EBP*β*, ACE2 and collagen I expression in angiotensin II–treated cardiac fibroblasts. (a–c) Representative Western blots and quantitative analysis of collagen I, angiotensin‐converting enzyme 2 (ACE2) and CCAAT/enhancer‐binding protein‐*β* (C/EBP*β*) protein levels in cardiac fibroblasts (CFs) treated with angiotensin II (Ang II, 1 μM). CFs were divided into three groups: (1) control (LV‐NC + si‐NC); (2) LV‐C/EBP*β* + si‐ACE2; and (3) LV‐C/EBP*β* + si‐NC. *β*‐tubulin was used as the loading control. Data are expressed as the mean ± standard deviation (SD) of three independent experiments. Statistical analysis was performed using one‐way analysis of variance (ANOVA) followed by Fisher's least significant difference (LSD) post hoc test. *p < 0.05 versus LV‐NC + si‐NC; ^#^ *p* < 0.05 versus LV‐C/EBP*β* + si‐NC.

Compared with the LV‐NC + si‐NC group, ACE2 expression was significantly increased, whereas collagen I expression was markedly reduced in the LV‐C/EBP*β* + si‐NC group (*p* < 0.05; Figure 9a,b). In contrast, relative to the LV‐C/EBP*β* + si‐NC group, ACE2 expression was clearly decreased and collagen I expression was concomitantly increased in the LV‐C/EBP*β* + si‐ACE2 group (*p* < 0.05; Figure 9a,b). Overall, these findings support a mechanism whereby C/EBP*β* attenuates collagen production through the induction of ACE2.

## 4 Discussion

Accumulating evidence has shown that dysregulation of the RAS, particularly elevated Ang II, plays a pivotal role in hypertensive cardiac disease and myocardial fibrosis. In this study, we found that C/EBP*β*, a transcription factor, is significantly downregulated in the myocardium of SHR rats. Overexpression of C/EBP*β* ameliorated hypertensive cardiac remodelling by modulating the ACE2/Ang‐(1–7) axis. Indeed, overexpression of C/EBP*β* significantly elevated myocardial ACE2 expression and Ang‐(1–7) levels while decreasing ACE and Ang II, which was associated with a reduction in systolic and diastolic dysfunction and attenuation of myocardial hypertrophy and fibrosis. To further substantiate the protective role of C/EBP*β*, we demonstrated that restoring C/EBP*β* expression mitigated hypertension‐induced myocardial fibrosis and ventricular remodelling. Taken together, these data indicate that increasing C/EBP*β* expression mitigates myocardial fibrosis in hypertensive cardiac disease by upregulating the cardioprotective ACE2/Ang‐(1–7) pathway, providing a novel therapeutic strategy.

Ang II promotes hypertensive cardiopathy and nephropathy by directly activating TGF‐*β*/Smad3 signalling and indirectly via TGF‐*β*/ERK/p38 MAPK‐Smad crosstalk [18, 19, 20, 21]. As a carboxypeptidase, ACE2 catalyses the hydrolysis of Ang II to generate the heptapeptide Ang‐(1–7). This metabolite exerts RAS‐modulating effects that counter‐regulate Ang II, the primary effector of the RAS [22, 23]. The ACE2/Ang‐(1–7) axis has emerged as a key protective mechanism in hypertension‐related cardiac injury; for instance, experimental interventions that enhance ACE2 expression have been shown to attenuate fibrosis and improve ventricular function by counterbalancing the deleterious effects of Ang II [24]. Beyond antagonising Ang II/AT1R‐mediated signalling pathways via the Mas receptor, Ang‐(1–7) potentiates bradykinin‐induced activation of the B2 receptor, thereby promoting nitric oxide‐dependent vasodilation. Importantly, these mechanisms operate largely independently of changes in blood pressure to inhibit Ang II‐mediated myocardial hypertrophy and fibrosis [25, 26]. Our experimental data showed that C/EBP*β* overexpression upregulated ACE2 expression, thereby reducing Ang II and increasing Ang‐(1–7) levels in SHR rats. Western blot and immunohistochemical analyses confirmed significant decreases in myocardial TGF‐*β*, collagen I, and collagen III levels in the C/EBP*β*‐overexpressing group. Echocardiography further revealed substantial improvement in ventricular hypertrophy and cardiac function compared to baseline measurements. These findings collectively validate our hypothesis that enhanced C/EBP*β* expression attenuates myocardial fibrosis and improves cardiac function in hypertensive cardiac disease via ACE2 upregulation. In line with these in vivo observations, our complementary in vitro experiments in Ang II‐stimulated primary cardiac fibroblasts showed that ACE2 knockdown markedly blunted the inhibitory effect of C/EBP*β* overexpression on collagen I synthesis, providing direct mechanistic evidence that C/EBP*β* attenuates collagen production, at least in part in an ACE2‐dependent manner.

As a pleiotropic cytokine, Ang II can trigger inflammatory cascades and oxidative stress responses [27]. These mechanisms directly mediate vascular and myocardial injury, with substantial evidence demonstrating that Ang II‐initiated cardiac inflammation accelerates fibrotic progression and cardiac remodelling [28, 29]. Through suppression of inflammatory responses, Lycorine ameliorates hypertension‐induced cardiac fibrosis and remodelling by targeting the PI3K/Akt/NF‐κB signalling pathway [30]. Elabela, by modulating the IL‐6/STAT3/GPX4 pathway, attenuates Ang II‐induced ferroptosis in cardiac microvascular endothelial cells, thereby suppressing adverse myocardial remodelling and fibrosis in hypertensive cardiac disease [31]. Endoplasmic reticulum (ER) stress inducers markedly upregulate C/EBP*β* expression in glomerular mesangial cells, thereby suppressing TNF‐*α* or IL‐1*β*‐triggered NF‐κB activation independently of its transactivation function. Specifically, C/EBP*β* overexpression inhibits NF‐κB activation by modulating IκB*α* degradation, revealing a direct molecular mechanism underlying the regulation of inflammatory signalling. This is functionally substantiated by the complete reversal of ER stress‐mediated NF‐κB suppression upon siRNA‐mediated knockdown of C/EBP*β*, confirming its central regulatory role in attenuating glomerular inflammation. Our study demonstrates that C/EBP*β* overexpression alleviates hypertension‐induced inflammatory cascades by significantly suppressing key inflammatory cytokines, including IL‐6 and MCP‐1 [32]. This anti‐inflammatory action thereby provides a mechanistic basis for the subsequent reduction of cardiac fibrosis and structural remodelling in hypertensive pathology.

Multiple studies indicate that C/EBP*β*, a basic leucine zipper transcription factor, participates in cardiac structural remodelling [11, 12]. Research suggests that C/EBP*β* binds to serum response factor (SRF) and, via SRF, associates with the promoter of zinc finger transcription factor 4 (GAtA4) to regulate GAtA4 expression, thereby influencing cardiomyocyte differentiation and other biological functions [13]. The histone deacetylase inhibitor CG200745 attenuated cardiac hypertrophy in DOCA‐induced hypertensive rats by inhibiting mTORC1 signalling via C/EBP*β*/TSC2 pathway activation. CG enhanced C/EBP*β* acetylation, promoting its TSC2 promoter binding and subsequent TSC2 upregulation, thereby suppressing mTORC1 activity and reducing left ventricular remodelling, cardiomyocyte hypertrophy and cardiac dysfunction [33]. In this study, we observed significantly elevated levels of Ang II and ACE alongside reduced levels of ACE2 and Ang‐(1–7) in the myocardial tissue of SHR rats. Conversely, in SHR rats subjected to C/EBP*β* overexpression, increased C/EBP*β* expression was accompanied by elevated ACE2 and Ang‐(1–7) content, while Ang II and ACE levels decreased. These data suggest that C/EBP*β* directly or indirectly modulates the expression of key RAS components in the hypertensive myocardium. Based on our previous experimental findings, C/EBP*β* has been shown to bind to the promoter region of ACE2 under Ang II stimulation, thereby regulating atherosclerotic plaque stability and disease progression. Similarly, under hyperglycaemic conditions, C/EBP*β* binding to the ACE2 promoter modulates myocardial fibrosis and cardiomyocyte apoptosis in diabetic cardiomyopathy [14]. Therefore, we demonstrate that in hypertensive cardiac disease, C/EBP*β* regulates ACE2 expression, consequently influencing other components of the RAS and contributing to pathological remodelling and functional alterations characteristic of this condition. Our study thus identifies a potential therapeutic target for ameliorating cardiac dysfunction and remodelling in patients with hypertensive cardiac disease.

Notably, C/EBP*β* knockdown did not further aggravate myocardial fibrosis in SHR rats. One possible explanation is that myocardial C/EBP*β* expression was already markedly reduced in SHR rats compared with normotensive controls, which may limit the additional impact of further knockdown on collagen synthesis and cardiac remodelling. In addition, myocardial fibrosis is regulated by multiple, partially redundant transcriptional pathways and other pro‐fibrotic regulators may compensate for the loss of C/EBP*β*, thereby blunting the phenotype in the knockdown group [34, 35, 36]. Moreover, our observations were obtained at a single disease stage in SHR rats, so it is possible that we missed a critical time window during which C/EBP*β* loss exerts a stronger effect. Taken together, these considerations suggest that C/EBP*β* may act as an important, but not exclusive, modulator of hypertensive cardiac remodelling.

This study has several limitations that should be considered. First, the exclusive reliance on the SHR rat model limits direct translational applicability to human pathophysiology. While this model reproduces key features of hypertensive cardiac remodelling, interspecies differences in RAS regulation and fibrosis‐related signalling pathways warrant caution when extrapolating these mechanistic findings to clinical settings. Second, lentiviral vectors were not used in WKY rats, resulting in an asymmetric control design. Nevertheless, our mechanistic conclusions regarding C/EBP*β* are drawn from comparisons among SHR groups that all shared the same lentiviral backbone, with the SHR + LV‐NC group serving as the empty‐vector control. Third, the experimental design primarily relied on lentiviral gene delivery to modulate C/EBP*β* expression rather than on pharmacologically tractable interventions, which limits the immediate translational therapeutic relevance of these findings.

## 5 Conclusion

Our study shows that myocardial C/EBP*β* expression is diminished in hypertensive rats and that restoring this transcription factor markedly limits hypertension‐induced cardiac injury. Augmentation of C/EBP*β* reduced arterial pressure, improved both systolic and diastolic performance, and mitigated left ventricular hypertrophy, interstitial fibrosis and inflammatory activation. At the molecular level, C/EBP*β* enhanced the ACE2/Ang‐(1–7) axis while restraining the classical ACE/Ang II pathway, a shift that was associated with reduced collagen accumulation and attenuation of adverse ventricular remodelling. These findings support C/EBP*β*‐driven reinforcement of ACE2 signalling as a potential approach to prevent or slow the progression of hypertensive cardiac remodelling.

## Author Contributions

**Bincheng Ren:** data curation. **Zehao Lin:** data curation, investigation. **Yuanyuan Tie:** conceptualization, funding acquisition, methodology, writing – original draft, project administration, resources. **Donggang Han:** supervision, resources. **Ming Zhang:** formal analysis, software. **Kexin Li:** formal analysis, software. **Ping Jin:** visualization.

## Funding

This study was supported by the Natural Science Foundation of Shaanxi Province (2022JQ‐886).

## Conflicts of Interest

The authors declare no conflicts of interest.

## Data Availability

The data that support the findings of this study are available from the corresponding author upon reasonable request.
